# Invar/WC Composite Compacts Obtained by Spark Plasma Sintering from Mechanically Alloyed Powders

**DOI:** 10.3390/ma15196714

**Published:** 2022-09-27

**Authors:** Călin-Virgiliu Prica, Traian Florin Marinca, Bogdan Viorel Neamțu, Argentina Niculina Sechel, Florin Popa, Elekes Marton Jozsa, Ionel Chicinaș

**Affiliations:** 1 Materials Science and Engineering Department, Faculty of Materials and Environmental Engineering, Technical University of Cluj-Napoca, 103-106, Muncii Ave, 400641 Cluj-Napoca, Romania; 2 Universal Alloy Corporation, Dumbravita 244A, 437145 Maramures, Romania

**Keywords:** Invar/WC composite compacts, spark plasma sintering, coefficient of thermal expansion

## Abstract

The composite materials are used on an increasingly large scale in top fields, such as the automotive, aerospace, and nuclear industries, due to the combination of the specific properties of the composite components. Invar/WC nanocrystalline composite compacts were successfully obtained by spark plasma sintering from mechanical milled composite powder. The influence of the amount of tungsten carbide (WC) on sintering, coefficient of thermal expansion (CTE), and hardness has been investigated. The relative density and hardness of Invar/WC composite compacts increases with increasing the WC content up to 10 vol.%. At higher amount of WC (15% vol.), the relative density and hardness of the Invar/WC composite compacts decreases. The temperature up to which CTE remains at a low value (0.6–1) × 10^−6^ °C^−1^ is influenced by the WC content and decreases with the WC amount of increase.

## 1. Introduction

The Invar effect in Fe–Ni alloys with face-centered cubic (fcc) structures, which have Ni concentrations around 35%, was discovered by Guillaume in 1897 [1]. The Invar’s coefficient of thermal expansion (CTE) at the ambient temperature is less than 2 × 10^−6^ °C^−^^1^, as compared with the coefficient of thermal expansion of most metals, which is between (10−20) × 10^−6^ °C^−1^ [2,3]. Due to their properties, the Invar-type alloys are used in high precision mechanical instruments, large size cryogenic liquid containers, large telescopes, electronic industry, or aerospace technology [4,5,6]. A disadvantage of the Invar alloys is that it has both low mechanical strength and hardness, due to its crystalline structure. The Invar alloys obtained by classical technology, melting, and casting, have a low strength of about 550 MPa [7,8]. To improve the strengthening and hardness, some researchers suggest crystallites refinement, precipitation hardening of carbides, or intermetallic compounds, etc. [9,10,11,12].

The synthesis techniques, specific to powder metallurgy, are often used for Invar structure refinement, as well as to homogenous dispersion of metallic carbides in Invar matrix. Invar in powder form can be obtained by mechanical alloying (MA), starting from elemental powders of Fe and Ni [10,13,14]. Additionally, the MA is used to obtained composite powders from metals whose equilibrium diagram does not show solubility, such as the W-Cu system [15]. By this technique, the crystallite sizes reach dimensions nanometers order. In order to preserve these dimensions, the powders can be compacted by spark plasma sintering (SPS) process [16,17]. The tensile strength obtained by sintering and plastic deformation, such as hot forging of the Invar-10WC composite, reach 1050 MPa after cold working [18]. Additionally, some authors reported a value of tensile strength for Invar up to 915 MPa by reducing the crystallite sizes in nano range [19]. The strengthening of Invar by precipitation of Ti and Cr carbides also was investigated [4,20]. The effect of Ti and V on hardness and tensile strength of Invar type alloy has been studied [21]. It has been reported that the tensile strength of Invar can be increased up to 650 MPa by precipitation of Cr carbides [4]. Some studies have shown that the strengthening of Invar alloy through carbide-forming elements, Ti, V, Zr, Nb, and Ta, achieved 1100 MPa tensile strength after heat treatment [22,23]. The strengthening of Invar alloys can be improved by precipitation of some intermetallic compounds, such as Ni_3_Ti, Ni_3_Al, Ni_4_Mo, and by Al or Mo alloying [24,25].

The effect of alloying with elements that combine with Ni from Invar is that the low CTE of Invar alloy increases because the Ni content in the Invar matrix decreases. Ni being the element that stabilizes the FCC structure and contributes to the low value of CTE and, to the magnetization of Invar type alloys [26].

The aim of this work is to obtain of an Invar/WC type composite compacts by spark plasma sintering from mechanically alloyed powders and its characterization. Our approach is to introduce WC alongside of Invar, in order to increase the mechanical characteristics of Invar. The main characteristics of tungsten carbide are its exceptional hardness, wear resistance, high modulus of elasticity, and maintenance of its room temperature hardness at higher temperatures, therefore recommending it use as a reinforcing compound in the metal matrix composite.

## 2. Experimental

Three types of nanocomposite Invar/WC powders were obtained by mechanical milling (MM). In the first stage, the Invar nanocrystalline powders were prepared by mechanical alloying technique (MA), as described in the Ref. [10]. Commercial carbide powder with particle size less than 100 μm, as raw material was used. In the second stage, the Invar/WC composite powder was obtained by milling. Invar nanocrystalline powder was mixed and homogenized with 5, 10, and 15 vol.% WC commercial powder. The mixtures were milled in argon atmosphere for 15 min in a Fritsch Pulverisette 6 (FRITSCH GmbH, Oberstein, Germany) high-energy ball mill. The Invar/WC composite powders were densified by SPS in a home-made equipment, in order to conserve the nanocrystalline structure in the sintered compacts. Home-made SPS equipment worked with a pressure of 30 MPa, and the current pulse had a rectangular shape. The intensity of the current pulse was 2000 A. The sintering temperature was measured at 2 mm from sample with a thermocouple inserted in graphite mould. Sintering parameters used for compacts synthesis were: sintering temperature in the range 700–900 °C, dwelling time at sintering temperature 0 min (without holding time), the pressure applied throughout the sintering process was 30 MPa, and the sintering atmosphere - Ar. An Inel Equinox 3000 diffractometer (INEL, Artenay, France) with Co radiation (λ_Co Kα_ = 0.1790300 nm) was used for the X-ray diffraction studies. The diffraction patterns were recorded in the angular range 2θ = 40–110°. The acquisition was performed in one step in the entire 2 theta range, specific for INEL diffractometer. The exposure time for each diffraction was 10 min. The Scherrer method was used for crystallite size determination. The resolution of the diffractometer was determined from the diffraction pattern of a reference sample. The particle size distribution has been determined using a Laser Particle Size Analyzer—Fritsch Analysette 22 Nanotec (FRITSCH GmbH, Oberstein, Germany), with an analysis field of 0.1–1000 µm. A JEOL-JSM 5600 LV (JEOL USA, Inc., Pleasanton, CA, USA) scanning electron microscope (SEM) coupled with an energy-dispersive X-ray (EDX) spectrometer (Oxford Instruments UltimMax65, Bognor Regis, UK) was used for the investigation of particles morphology, compact microstructures, and local chemical homogeneity. Dilatometry measurements were taken by an Ulbricht-Weiss dilatometer. The measurement method of density was the following. The SPS cylindrical samples were weighed with a laboratory balance, in order to find out its mass. The volume of sample was computed. The density was measured as the ratio between the mass and volume of the samples. The relative density was computed as the ratio between its density and the theoretical density of the composite compacts. The hardness was determined with Brinell hardness tester equipment, using a 5 mm diameter ball as a penetrator.

## 3. Results and Discussions

**Figure 1:**
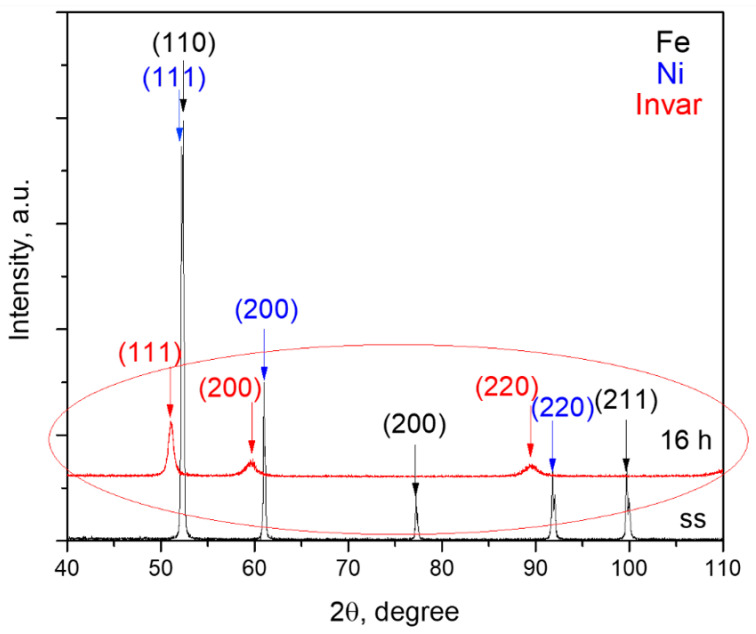
XRD patterns of Invar powder milled up to 16 h. SS refers to the starting sample. On the top of the figure are indicated the peaks positions for the indicated phases. For clarity the patterns are vertically shifted (after Ref [10]).

In the first stage, the Invar nanocrystalline powders were prepared by mechanical alloying technique (MA) from Fe and Ni elemental powders 16 h milled, as described in the Ref. [10], XRD patterns of Invar powder 16 h milled are shows in Figure 1, encircled for a better view. Only the Invar’s characteristic maxima were identified. The composite powder was obtained in stage two, from Invar powder mechanically alloyed up to 16 h, to which 5, 10, and 15 vol.% WC (commercial powder) was added.

**Figure 2:**
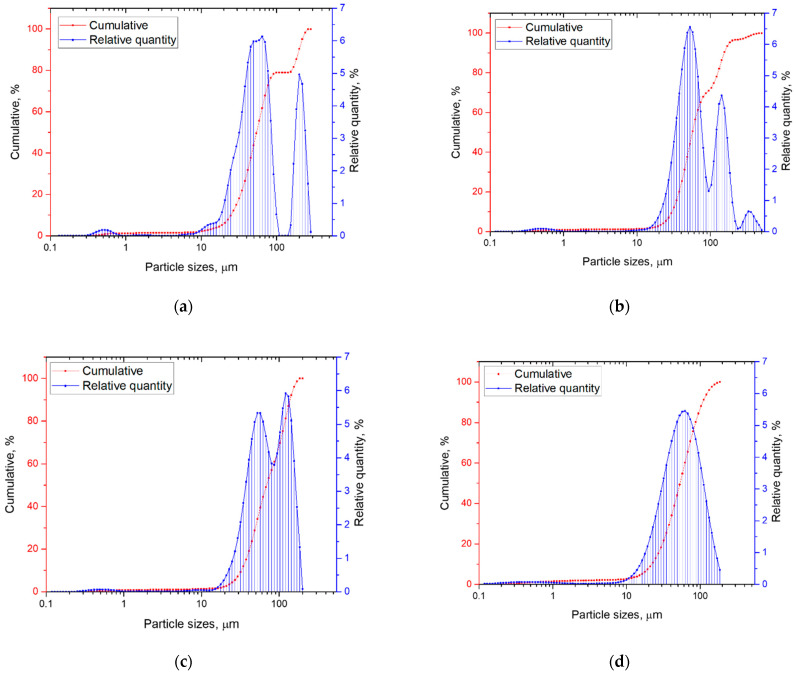
Particle sizes distribution of Invar powder milled 16 h—(**a**); Invar + 5% WC powder-(**b**); Invar + 10% WC-(**c**); Invar + 15% WC-(d). The Invar/WC mixture were 15 min milled.

The influence of WC addition on the particle size of Invar/WC composite powder was investigated. The particle sizes distributions of both the Invar powder milled 16 h and Invar/WC composite powders after 15 min milling are shown in Figure 2. The mixture of Invar + WC powders were subjected to mechanical milling for 15 min, in order to increase the dispersion of tungsten carbide particles (−20 µm, D50 = 2 µm) and create the composite Invar/WC particles. It can be observed that the quantity of WC powder from composite powder influences the size distribution of particles. Invar powder has a wide (5–300 µm) polymodal particle size distribution with two maxima centered at 60 and 180 µm (Figure 2a). Additionally, the presence of a small fraction of particles smaller than 1 µm can be observed. In the case of 5 vol.% WC, adding in the mechanical milled mixture (Figure 2b), it is facilitated the agglomeration of some particles that were present in the composite mixture, as well as particles with larger dimension, between 200 and 500 µm, in a proportion of 4%. With WC quantity increasing (Figure 2c,d), the maximum value of the particles from composite powders shifted to smaller values. The distribution curve of Invar + 10% WC composite powder becomes bimodal and cvasi-Gaussian for Invar + 15% WC powder.

The particles size characteristics are summarized in Table 1.

The values of D10, D50, and D90 show that the D50 increase with WC added up to 10%, due to the WC particles covering the Invar particles, which leads to the growth of composite powder particles. Increasing the amount of WC (15 vol.%) favors particle fragmentation, with tungsten carbide being hard and brittle.

**Figure 3:**
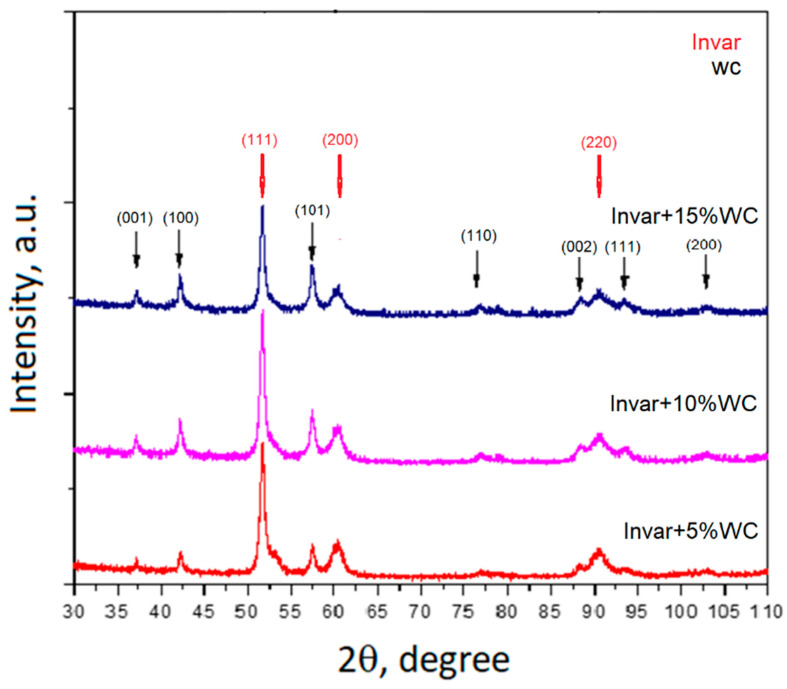
XRD patterns of Invar/WC mixtures—mechanically milled for 15 min.

The XRD pattern of Invar/WC mixtures are shown in Figure 3. For all mixtures, only Invar and WC maxima were identified. This indicates that no reactions occurred during milling process, resulting in Invar/WC composite powders. Additionally, the intensities of the WC peaks increase proportionally with its addition in the composite powders, as expected.

**Figure 4:**
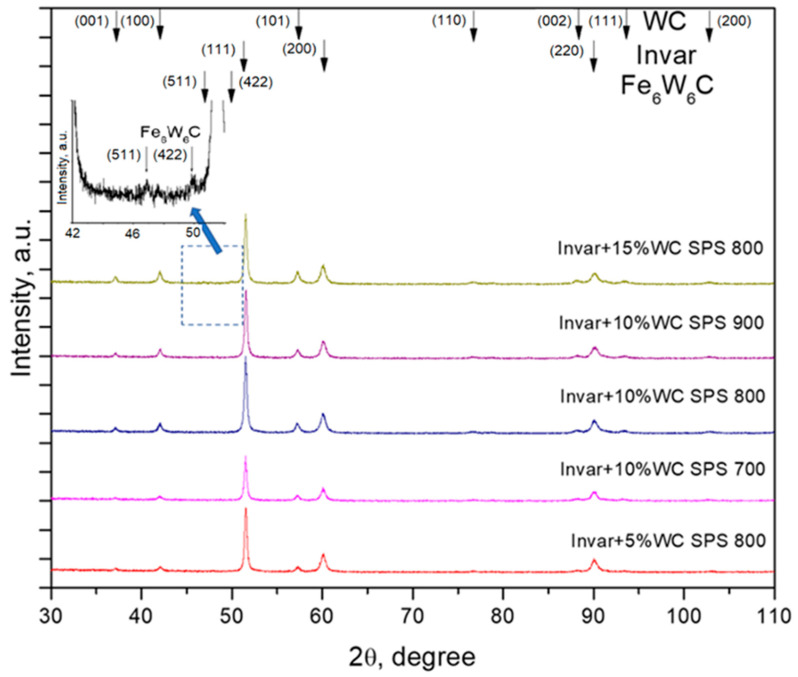
XRD diffraction patterns of Invar/WC compacts sintered at different temperatures.

From Invar/WC composite, powders were made compact by SPS technique. The XRD diffraction patterns of Invar/WC compacts at different sintering temperatures are shown in Figure 4. It can be noted that the Invar’s characteristic maxima became narrowed as the sintering temperature increased, due to removal of internal stresses that were induced by mechanical milling process, as well as the increase of the crystallite sizes. Crystallites increase due to recrystallization during spark plasma sintering. However, the growth of Invar crystallites is limited, due to WC particles, which are homogenously distributed at the grain’s boundary. Additionally, the crystallites growth is limited by short time of maintaining at sintering temperatures (0 min).

Is worth noting that, in the XRD patterns of Invar + 15% WC compacts obtained by SPS at 800 °C, the Fe_6_W_6_C characteristics peaks can be identified. A detail of the maxima positions for this phase is given in the figure. The mixed carbide (Fe_6_W_6_C) is form in situ, in a solid-state reaction between the Fe from Invar and WC, due to both high content of WC (15 vol.%) from the composite powder and high sintering temperature (800 °C). High amount of WC creates a larger reaction interface between Invar and WC, highlighting the formation of the new phase. It is possible to also have this phase in the case of the other compositions; however, due to the smaller interface, the amount of this phase is under the limit of the XRD technique.

In Table 2, the densities and relative density of Invar/WC composite compacts, as a function of WC amount and sintering temperature, are shown.

The relative density of Invar/WC composite compacts decreases with WC amount increasing at the same sintering temperature (800 °C). This is due to the presence of the very fine (<5 μm) and hard WC particles in an increasing amount, which reduce the pressability of the Invar/WC composite powders during SPS. Additionally, is known that the WC has a high electrical resistivity, compared to Invar. In such a case, a large amount of the current injected by the SPS equipment will pass through punches and mould (heating the mould and punches), and a lower amount of current will pass through the powder subjected to sintering. This essentially contributes to the decrease in densification, with WC amount increasing in the case of spark plasma sintering process. When the WC content is the same in all Invar/WC composite samples, the density increases as the sintering temperature increases, due to the increase of the diffusivity.

**Figure 5:**
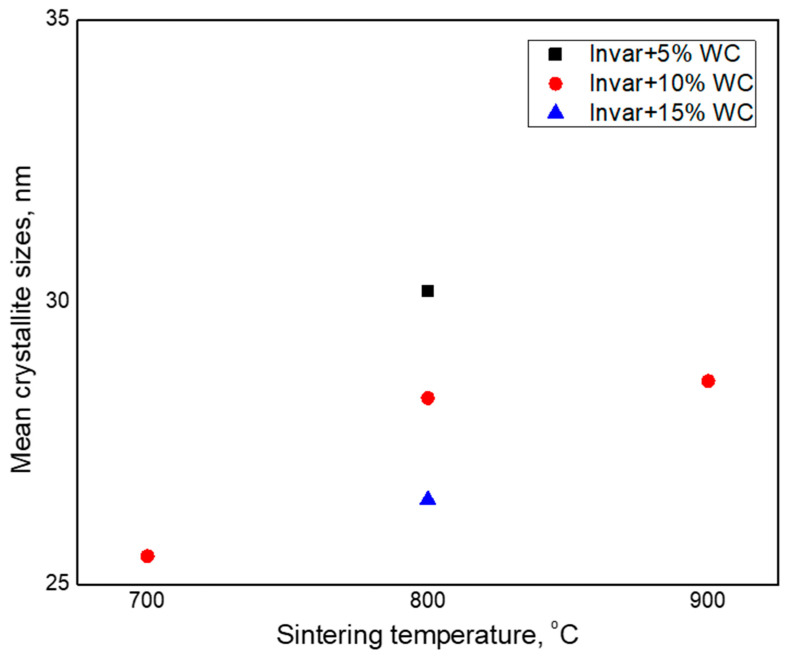
Mean crystallite sizes of Invar/WC composite compacts versus sintering temperatures.

The mean crystallite sizes of Invar phase versus sintering temperature are shown in Figure 5. For compacts obtained from Invar + 10% WC, we can conclude that the increase of the sintering temperature leads to an increase of size Invar crystallite. Thus, the mean crystallite size of Invar from the sintered compacts at 700 °C increases from 25 to 28 nm in the case of sintered compacts at 800 °C, and it reaches 29 nm when the sintering temperature is 900 °C. Additionally, the crystallite sizes of composite compacts with different amount of WC, sintered at the same temperature (800 °C), become smaller as the carbide content increases. So, for the Invar + 5% WC compact, the average crystallite size is 30 nm. When the WC content increases to 10%, the crystallite size decreases to 28 nm and reaches 27 nm at 15% WC. For comparation, the average crystallite size of Invar powder obtained after 16 h of milling was 17 nm. The WC has a high electrical resistivity, which influences the resistivity of the composite powder; as a result, the plasma sintering process is difficult. For this reason, the energy transferred to the powder by electrical discharge decreases, and this led to the lower increase of crystallites. The energy transferred to the powder by electrical discharge as heat. The growth of crystallites occurs due to the recrystallization phenomena that occurs at high temperatures (0.4 of the melting temperature). Due to the lower energy transferred to the powder, the temperature is also low, and recrystallization occurs incompletely. Combined with spark plasma sintering, without holding time at sintering temperature, crystallite growth is limited.

**Figure 6:**
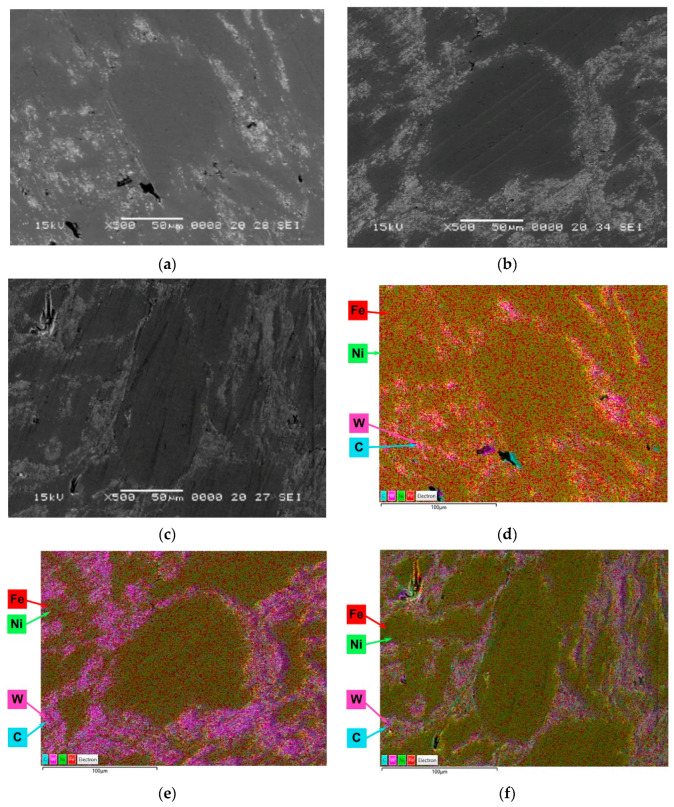
SEM images and EDX elemental maps distribution of Invar/WC composite compacts sintered at 800 °C—(**a**) and (**d**) Invar + 5% WC, (**b**) and (**e**) Invar + 10% WC, and (**c**) and (**f**) Invar + 15% WC.

The SEM images and EDX elemental maps distribution of the Invar/WC SPS compacts sintered at 800 °C are shown in Figure 6. In the SEM images of Invar + 5% WC (Figure 6a), Invar + 10% WC (Figure 6b), and Invar + 15% WC (Figure 6c), for composite compacts sintered at 800 °C, the presence of two types of phases can be observed: the Invar particles (dark area) surrounded by fine WC particles (light area). The map distribution of the elements (Figure 6d–f) shows that the WC particles are small (<1 μm) and evenly distributed around the Invar particles. Invar particle size varies in the range 10–150 μm.

**Figure 7:**
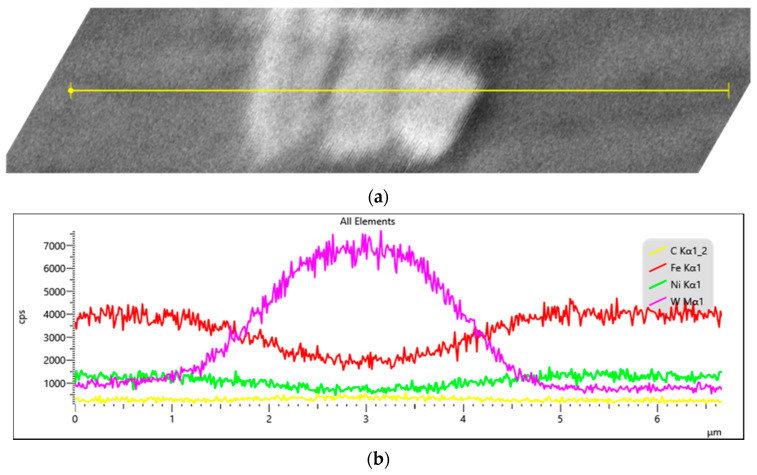
SEM image—(**a**) EDX line scan analyze; (**b**) WC particle embedded in Invar matrix.

Figure 7a shows the SEM image of a WC particle embedded in an Invar matrix. Elemental concentration profiles of W, Fe, Ni, and C across the yellow line are exposed in Figure 7b. It can be observed that the decrease of the W content is not sudden at the edge of the WC particle. The iron content has an opposite tendency. Therefore, it can be concluded that there is a mutual diffusion at the interface, both of W in the Invar metal matrix and of Fe in the WC particle. The diffusion distance is about 1 μm. We assume that the Fe_6_W_6_C chemical compound was formed in the interface area, as also highlighted by XRD analyses.

**Figure 8:**
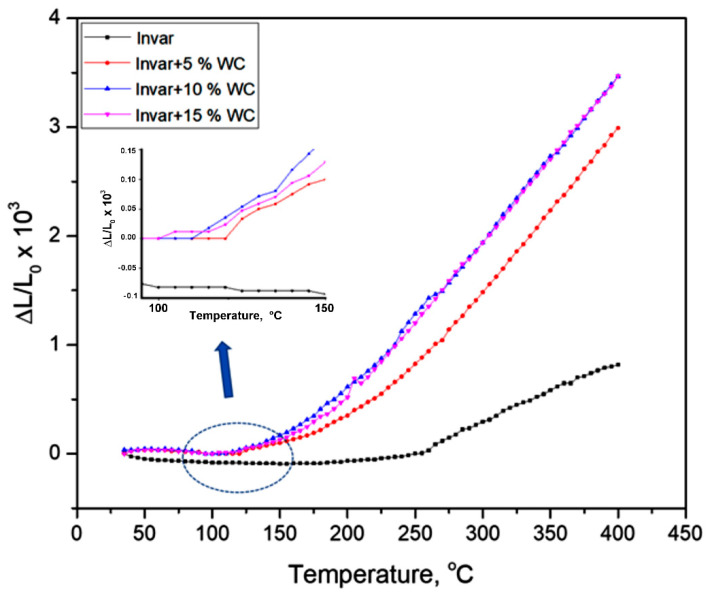
Δl/L_0_ versus temperature plots of Invar (■), Invar + 5% WC (●), Invar + 10% WC (▲), and Invar + 15% WC (▼).

The sintered samples of Invar + 5% WC, Invar + 10% WC, and Invar + 10% WC compacts were heated up to 400 °C, in order to determine the coefficient of thermal expansion. For all samples, the sintering temperature was 800 °C. The expansion (Δl/L_0_) curves of both the Invar and Invar/WC composite compacts versus temperature are shown in Figure 8.

From Figure 8, it can be noticed that the thermal expansion curve of Invar compact sample has a very slight increase up to 250 °C. In the range 250–400 °C, Δl/L_0_ proportionally and continuously increase with temperature increasing. The slope of the curve changes suddenly from 250 °C.

For Invar/WC composite compacts the Δl/L_0_ curve versus temperature presents an invariable zone with temperature, lower than in the case of Invar, the inflection point being at lower temperatures as the WC content increases. Thus, the inflexion points of Δl curve of Invar + 5% WC corresponds to 120 °C. For Invar + 10% WC, it is at 105 °C; for Invar + 15% WC, it is up to 100 °C. From the inflection point, the increase of Δl/L_0_ is more rapid and continuous up to 400 °C for all Invar/WC sintered samples; the slope of the curves is higher than that of the curve corresponding to the Invar sintered sample.

The CTE values of Invar and Invar/WC sintered composite compacts are provided in Table 3. CTE was computed with the following relationship [27]:

(1) $$\alpha =\frac{1}{l_{o}}\times \frac{\Delta l}{\Delta T}$$

*l*_0_—initial length of the sample, Δ*l*—sample elongation, and Δ*T*—the temperature range.

It is known that the inflexion point of Δl = f(T) of Invar corresponds to its Curie temperature [13,28]. The Invar’s Curie temperature is 528 K (255 °C) [29]. The calculated value of CTE for the Invar36 compacts, up to 250 °C (0.6 × 10^−6^ °C^−1^), agrees with the CTE of Invar36 bulk alloy obtained by classic technologies [2,3,30]. In the case of the Invar/WC sintered composite compacts, the CTE value is 1 × 10^−6^ °C^−1^, regardless of WC content, up to the inflexion point. This value is 67% higher than the CTE value of Invar sintered compacts, due to the higher CTE value of WC [31]. It can be observed that at temperatures higher than their inflexion points, the CTE of both Invar and Invar/WC nanocrystalline compacts significantly increases. With the increase in the amount of WC, the value of CTE also increases, since the expansion of the WC is higher than that of the Invar at high temperatures [17]. Additionally, the Fe_6_W_6_C chemical compound, which is formed at the interface between WC and Invar particles, influences the increase of CTE with the increase of temperature. The CTE of Fe_6_W_6_C is about 7 × 10^−6^ °C^−1^ in the range of temperature 400–1200 K [32].

One of the purposes of reinforcing the Invar with WC was to increase the mechanical properties, especially the hardness of the Invar/WC composite compacts. The hardness was determined by the Brinell method. A 5 mm diameter carbide ball was used as a penetrator, and 31.5 kg was the load. The hardness values are given in Table 4. For a better understanding of the WC added in Invar, as a reinforcement element on the hardness of composite compacts, the relative density values of the sintered samples were also entered in Table 4.

It is very known that the hardness is influenced both by the hardness of the constituents and relative density (porosity) of alloy. Due to the crystalline structure lattice of Invar (FCC), its hardness is low. When increasing the temperature of sintering from 700 °C to 900 °C, it can be noticed that the hardness of Invar/WC composite compacts with the same WC content (10 vol.%) also increase. This increase in hardness can be explained only by an increase in relative density. Therefore, the hardness of Invar + 10% WC sintered at 700 °C is 32 HB; it is 64 HB when the sintering temperature is 800 °C, and it reaches the value of 73 HB when sintering was performed at 900 °C. Thus, even a 6% increase in relative density leads to a 130% increase in hardness. If the SPS is performed at the same temperature (800 °C), it can be seen that the hardness of the Invar/WC composite compacts is twice as high (67 HB compared to 36 HB) if the WC content increases from 5 to 10%, provided that the relative densities are close (83 versus 79%). On the other hand, even if the amount of carbide increases from 10 to 15%, the hardness increases insignificantly, from 64 to 67 HB, due to the decrease of the relative density from 78.6 to 71.2%.

## 4. Conclusions

Invar/WC composite compacts was successfully obtained by spark plasma sintering from Invar and WC powders. The mean crystallite sizes of Invar from composite compacts are maintained in the nanoscale range. The relative density of Invar/WC composite compacts syntheses by SPS decreases with WC amount. It increases at the same sintering temperature, due to the hardness and high electrical resistivity of the WC particles, compared with the Invar.

The calculated value of CTE for the Invar compacts, up to 250 °C, is 0.6 × 10^−6^ °C^−1^. In the case of the Invar/WC sintered composite compacts, the CTE value is 1 × 10^−6^ °C^−1^, up to 120 °C, at a content of 5% WC. This value remains constant up to 105 °C with the increase of the WC content to 10% and up to 110 °C if the WC content increases to 15%.

The hardness of Invar/WC composite compacts is influenced by the WC quantity and relative density of sintered compacts. Upon increasing the sintering temperature from 700 to 900 °C, the hardness of Invar/WC composite compact with the same WC content (10 vol.%) increased from 32 HB to 73 HB, respectively. If the hardness of Invar/WC composite compacts sintered at the same temperature (800 °C), it doubles (67 HB versus 36 HB). The addition of WC amount increases from 5 to 10%, but the increase is insignificant (from 64 to 67 HB) if the amount of tungsten carbide increases from 10 to 15%, due to the decreasing of the relative density from 78.6 to 71.2%.

By increasing the WC content of Invar/WC composite compacts, the CTE decreases, but the hardness of compacts grows. A compromise is, thus, reached, in which the mechanical properties increase in the CTE damage. The temperature ranges, up to which this type of composite can be used successfully, decreases; however, the mechanical properties reach two times higher values.

## Figures and Tables

**Table 1:** The D10, D50, and D90 values of composite powders obtained after 15 min. of mechanical milling, as well as the ones of nanocrystalline Invar.

Composite Powder	D10 (µm)	D50 (µm)	D90 (µm)
Invar	24.8	54.3	199.4
Invar + 5% WC	29.8	56.8	154.6
Invar + 10% WC	32.7	68.8	138.6
Invar + 15% WC	22.1	53.2	108.2

**Table 2:** Values of density and relative density (compactness) of Invar/WC composite compacts.

Sample	Sintering Temperature (°C)	Density (g/cm^3^)	Relative Density (%)
Invar + 5% WC	800	7.45	83.8
Invar + 10% WC	700	7.29	76.2
Invar + 10% WC	800	7.52	78.6
Invar + 10% WC	900	7.58	83.4
Invar + 15% WC	800	7.29	71.2

**Table 3:** CTE values of Invar and Invar/WC sintered composite compacts.

Sample—Sintered at 800 °C	Temperature Range, ΔT (°C)	α × 10^6^ (°C^−1^)
Invar	20–250 250–400	0.6 10.0
Invar + 5% WC	20–120 120–400	1.0 10.6
Invar + 10% WC	20–105 105–400	1.0 11.9
Invar + 15% WC	20–100 100–400	1.0 12.3

**Table 4:** The hardness values of Invar/WC composite compacts sintered at different temperatures.

Sample	Sintering Temperature (°C)	Hardness (HB)	Relative Density (%)
Invar + 5% WC	800	36	83.8
Invar + 10% WC	700	32	76.2
Invar + 10% WC	800	64	78.6
Invar + 10% WC	900	73	83.4
Invar + 15% WC	800	67	71.2
